# Cortisol concentration affects fat and muscle mass among Polish children aged 6–13 years

**DOI:** 10.1186/s12887-021-02837-3

**Published:** 2021-08-27

**Authors:** Paulina Pruszkowska-Przybylska, Aneta Sitek, Iwona Rosset, Marta Sobalska-Kwapis, Marcin Słomka, Dominik Strapagiel, Elżbieta Żądzińska, Niels Morling

**Affiliations:** 1 Department of Anthropology, Faculty of Biology and Environmental Protection, University of Łódź, Banacha 12/16, 90-237 Łódź, Poland; 2 Biological Anthropology and Comparative Anatomy Research Unit, School of Medicine, The University of Adelaide, South Australia 5005 Adelaide, Australia; 3 The Biobank Lab, Department of Molecular Biophysics, Faculty of Biology and Environmental Protection, University of Lodz, Lodz, Poland; 4 BBMRI.pl Consortium, Wrocław, Poland; 5 Section of Forensic Genetics, Department of Forensic Medicine, Faculty of Health and Medical Sciences, University of Copenhagen, Frederik V’s Vej 11, 2100 Copenhagen, Denmark

**Keywords:** Body composition, obesity, cortisol level

## Abstract

**Background:**

Cortisol is a steroid hormone acting as a stress hormone, which is crucial in regulating homeostasis. Previous studies have linked cortisol concentration to body mass and body composition.

**Methods:**

The investigations were carried out in 2016–2017. A total of 176 children aged 6–13 years in primary schools in central Poland were investigated. Three types of measurements were performed: anthropometric (body weight and height, waist and hip circumferences), body composition (fat mass FM (%), muscle mass – MM (%), body cellular mass - BCM (%), total body water - TBW (%)), and cortisol concentration using saliva of the investigated individuals. Information about standard of living, type of feeding after birth, parental education and maternal trauma during pregnancy was obtained with questionnaires.

**Results:**

The results of regression models after removing the environmental factors (parental education, standard of living, type of feeding after birth, and maternal trauma during pregnancy) indicate a statistically significant association between the cortisol concentration and fat mass and muscle mass. The cortisol concentration was negatively associated with FM (%) (Beta=-0.171; *p* = 0.026), explaining 2.32 % of the fat mass variability and positively associated with MM (%) (Beta = 0.192; *p* = 0.012) explaining 3.09 % of the muscle mass variability.

**Conclusions:**

Cortisol concentration affects fat and muscle mass among Polish children.

**Trial registration:**

The Ethical Commission at the University of Lodz (nr 19/KBBN-UŁ/II/2016).

**Supplementary Information:**

The online version contains supplementary material available at 10.1186/s12887-021-02837-3.

## Background

Cortisol is a steroid hormone produced by the adrenal cortex, widely known as a stress hormone, crucial in the regulation of homeostasis. Cortisol acts as a neuroendocrine mediator of stress responses in organs and effector tissues such as the brain, cardiovascular system, immune system, fat, and muscle tissue [1]. The activation of intracellular glucocorticoid receptors leads to changes in the metabolism, structure, conduction of stimuli in the cell, and changes the expression of genes such as *ID2*, *C3*, and *OASI* [2]. Excess secretion of cortisol is observed in patients with Cushing Syndrome and healthy individuals during stress. Hypercortisolism is dangerous for the body and has an extremely wide spectrum of effects including insulin resistance, obesity, insomnia, hyperglycaemia, elevated cholesterol and triglycerides levels, decreased immune response, and tissue weakening of antioxidant enzyme activity [3].

Numerous studies of the association between body mass/proportion and stress level have been performed using questionnaires and measurements of cortisol concentration e.g. [4]. Regardless of the chosen method, the results were inconsistent. Elevated stress levels were observed among individuals with increased BMI [5], although not statistically significant in all cases [6]. Yu et al. (2020) [7] showed that children with obesity displayed significantly lower morning cortisol and higher evening cortisol than normal- weight children. Thus, the diurnal salivary cortisol rhythm is associated with childhood obesity. Dai et al. (2021) [8] showed that lower first-in-morning diurnal and increased night cortisol levels were associated with increased body fat.

Elevated stress levels were found to be associated with decreased body mass among children aged 7–18 years during the political transformation in Poland in the early 1990s [9]. Trauma during pregnancy increased the risk of body weight deficit in 7–10 years-old Polish children, whose prenatal development occurred during the political and economic transformation of 1990s [10].

Most studies of associations between body composition and cortisol concentration have focused on fat and muscle tissue. Cortisol has a catabolic impact on fat tissue, and chronically persistent high concentrations of cortisol affects lipolysis leading to the release of glycerol and free fatty acids. This effect can be a direct action of the hormone or a result of reduced glucose uptake of adipose tissues [11]. High cortisol concentrations affect protein and carbohydrate metabolism in muscle tissue. Elevated cortisol concentration increases the release of gluconeogenesis substrates from peripheral tissues leading to muscle weakness [12]. Physical exercise increases the cortisol concentration due to temporary oxygen stress [13]. Thus, paradoxically elevated concentrations of cortisol can occur among physically active individuals with muscle mass above average.

This study aimed to assess if the cortisol concentration is associated with the composition and proportion of Polish children stratified according to parental education, the standard of living, type of feeding after birth, and maternal trauma during pregnancy.

## Materials and methods

The investigations were carried out between December 2016 and December 2017. A total of 176 children in primary schools in central Poland (Lodz, approximately 700,000 citizens) were included in the study. However, due to the lack of questionnaires concerning four children and unsuccessful results of the saliva sample of one child, only results of investigations of 171 children (girls = 93, boys = 78) were included.

The anthropometric measurements of body weight and height, waist and hip circumferences were performed according to Martin’s method [14] by the staff of the Department of Anthropology of the University of Lodz. Using these measurements, body mass index (BMI = body mass (kg)/ body height^2^ (m^2^) and waist to hip ratio (WHR = waist circumference (cm)/hip circumference (cm)) were calculated. The body composition was assessed by the staff of the Biobank Laboratory of the University of Lodz including percent of fat mass - FM (%), the percent of muscle mass – MM (%), percent of body cellular mass - BCM (%), and percent of total body water - TBW (%) using the bioelectrical impedance vector analysis (BIA-101 ASE, Akern, Italy). Information about the standard of living, type of feeding after birth, parental education, and maternal trauma during pregnancy was obtained with questionnaires.

### Questionnaire information

 The parents of the children provided signed approval for the investigations and filled in questionnaires. The standard of living was divided into three categories, as were also applied in the previous studies [15–17]: (1) low standard of living (‘we live very poorly, insufficient resources for basic needs or we live modestly, we have to be very economical on a daily basis’s), (2) medium standard of living (‘we live on average, it is enough for us every day but we have to save on more serious purchases’), (3) high standard of living (‘we live well enough for us without many special savings, or we live very well - we can afford full luxury’).

Children that were breastfed (independently of duration) were included in the breastfed group.

The parental educational background was categorized as: (1) basic (8 years at obligatory primary school and, in some cases, further three years at vocational school), (2) secondary education (4–5 years at secondary school or bachelor degree) and (3) higher education (full university degree).

The information about breastfeeding was categorised as yes (breastfeeding for at least one month) or no.

The children’s mothers reported if they had experienced traumatic events during the pregnancy such as violence in the family, natural disasters, pedestrian injuries and death of family members, and this variable was treated as categories, i.e. yes or no.

### Saliva sample collection and preparation

Saliva samples were collected in sterile Falcon tubes (Nest Biotechnology). The children did not eat, drink, chew chewing gum, or brush teeth for the last 30 min before sampling. Cortisol has the highest physiological concentrations in the morning around 8 am and then slightly decreases during the day and evening [18]. The cortisol concentration in serum and saliva is notably higher in the morning (8–9 pm) than in the afternoon (4–5 am) [19]. Due to the diurnal variation in the cortisol concentration, the cortisol concentration was normalised according to the time of collection and/or sex, according to the equation: (n- x)/SD, where: N is the cortisol level, X is the mean cortisol level for the proper hour (8, 9, 10, 11, 12 pm or 1, 2 am) and/or sex (boy or girl), and SD – standard deviation for each group.

The normalised cortisol concentrations were divided into three groups: (1) low (below Q1 (lower quartile)), (2) medium (between Q1 and Q3), and (3) high (above Q3 (upper quartile)).

The saliva was collected between 8 am and 2 pm. Immediately after the collection, the samples were stored at 2–8 °C and, on the same day, frozen and stored at − 20 °C until the investigation, no longer than three months after collection [20]. Before testing, the samples were thawed and centrifuged at 2 000 g to separate the mucins according to the recommendations of the manufacturer of the ELISA kit (cf. below).

The cortisol concentration of the saliva samples was measured using an enzyme-linked immunosorbent assay (ELISA) kit (DRG® Salivary Cortisol ELISA (SLV-2930), USA).

Each sample was measured in duplicates. The assay included seven standard concentrations. A micro plate reader (SpectraMax i3, Molecular Devices) was used to measure the absorbance at 450 nm. The 4PL method was used to plot the standard curves using an open-source software (https://www.aatbio.com/tools/four-parameter-logistic-4pl-curve-regression-online-calculator/). The concentration range of the investigated samples was in agreement with information supplied by the kit producer. Intra-assay CVs were less than 10 %. Correlation between saliva and serum cortisol level is noticed but the concentration values are not equal, thus it should be considered during drawing the conclusions [21]. However, the study of El-Farhan [22] indicated that measuring the cortisol level in serum, urine and saliva are reliable to each other.

### Statistical analysis

The children were divided into groups according to the calendar age. The FM (%), MM (%), BCM (%), TBW (%), WHR, and BMI values were standardised according to age and sex. Z-score equivalents were used for the calculations. Additionally, WHO norm BMI for age (2007) was used to arrange the adequate range for underweight, normal weight, overweight and obesity (https://www.fantaproject.org/sites/default/files/resources/FANTA-BMI-charts-Jan2013-ENG_0.pdf). The Chi-squared Pearson test was used to evaluate the sex differences between underweight, normal-weight, overweight, and obese children.

The following variables were normally distributed: FM (%), MM (%), BCM (%), TBW (%), WHR and BMI values, while the cortisol concentration was not normally distributed (*p* < 0.05).

The Mann-Whitney U-test was used to evaluate the differences in cortisol concentration between children with normal and increased BMI.

To assess the differences between cortisol concentration and sex, the standard of living, type of feeding after birth, parental education, and maternal trauma during pregnancy, the Kruskal-Wallis or the Mann-Whitney U-test were used for the analysis of FM (%), MM (%), BCM (%), TBW (%), WHR. The ANOVA or nonparametric Kruskal-Wallis tests were applied to assess possible differences between the cortisol concentration (low, medium, and high) and the composition and proportion of the body. The post hoc Tukey’s HSD test was used to perform multiple comparisons of all pairs of means. Additionally, the Spearman test was applied to calculate the correlation between cortisol concentration and the composition and proportion of the body.

Stepwise forward multiple regression models were used to identify variables that were statistically significantly correlated with FM (%), MM (%), BCM (%), TBW (%), WHR, and BMI. The residues for each body component, BMI, and WHR were used to assess the correlation with the cortisol concentration.

The effect size for each test was performed using online calculator: (https://www.danielsoper.com/statcalc/calculator.aspx?id=48). Cohen’s d was applied for the Mann-Whitney U test and ANOVA in the case of multiple regression Cohen’s f^2^ was done.

The study was conducted according to the guidelines laid down in the Declaration of Helsinki. All procedures involving research study participants were approved by the Ethical Commission at the University of Lodz (nr 19/KBBN-UŁ/II/2016). Written informed consent was obtained from all the subjects’ parents.

## Results

Among all children, 77.19 % had normal BMI (including 43.86 % of girls and 33.33 % of boys), 0.58 % were underweight, the rest of the children with excess BMI 16.37 % were overweight (including 9.94 % of girls and 6.43 % of boys) and 5.85 % were obese (including 0.58 % of girls and 5.26 % of boys) (Table 1).

**Table 1:** Characteristic of the investigated group of children concerning BMI according to the WHO norm BMI for age (2007) stratified into sex

	Sex	WHO norm - normal weight	WHO norm - overweight	WHO norm - obesity	WHO norm - underweight		Chi^2 Pearson	p	
	Girls	75	17	1	0	93	9.901	0.019	
		56.82 %	60.71 %	10.00 %	0.00 %				
% column		80.65 %	18.28 %	1.08 %	0.00 %				
% row		43.86 %	9.94 %	0.58 %	0.00 %	54.39 %			
% total among girls	Boys	57	11	9	1	78			
		43.18 %	39.29 %	90.00 %	100.00 %				
% column		73.08 %	14.10 %	11.54 %	1.28 %				
% row		33.33 %	6.43 %	5.26 %	0.58 %	45.61 %			
% total among girls	Total	132	28	10	1	171			
% total		77.19 %	16.37 %	5.85 %	0.58 %				

The cortisol concentration was not statistically significantly different between boys and girls (Table 2).

**Table 2:** Cortisol concentration (ng/ml) for boys and girls

Variable	Sex	N	Mean	Median	Q1	Q3	SD.	Z	p
cortisol concentration	Girls	93	1.6935	0.9969	0.4079	2.1771	2.7625	0.3845	0.7006
	Boys	78	1.9514	0.9361	0.4452	2.3231	2.5542		
	Total	171	1.8112	0.9732	0.4175	2.2930	2.6648		

The cortisol concentration was higher among children from families with a high standard of living (mean=-0.200) than among children with a medium standard of living (mean=-0.354), H = 6.532; *p* = 0.038) (Table 3). The cortisol concentration was not statistically significantly influenced by differences in sex, parental education, maternal trauma during pregnancy, or breastfeeding (Table 3).

**Table 3:** Cortisol concentration after standardisation and environmental factors in Polish children

Cortisol concentration	Mean	N	SD	Q1	Median	Q3	H/Z	P
Maternal education								
Basic	-0.587	7	0.310	-0.864	-0.503	-0.341	5.340	0.069
Medium	-0.090	35	0.931	-0.553	-0.278	0.083		
Higher	0.020	129	0.933	-0.568	-0.238	0.180		
Paternal education								
Basic	-0.239	14	0.461	-0.604	-0.314	0.101	0.273	0.873
Medium	-0.014	66	0.990	-0.451	-0.242	0.095		
Higher	-0.005	91	0.924	-0.612	-0.266	0.083		
Standard of living								
Low	-0.084	9	0.492	-0.372	-0.255	0.055	6.532	0.038
Medium	-0.154	80	0.920	-0.621	-0.354	-0.009		
High	0.103	82	0.945	-0.472	-0.200	0.278		
Sex								
Girls	-0.066	93	1.327	-0.822	-0.003	0.841	-0.501	0.616
Boys	0.098	78	1.337	-0.748	-0.066	0.917		
Breastfeeding								
No	0.013	14	1.232	-0.612	-0.369	-0.159	0.662	0.508
Yes	-0.031	157	0.892	-0.568	-0.249	0.095		
Trauma during pregnancy								
No	-0.005	154	0.941	-0.553	-0.241	0.101	1.298	0.194
Yes	-0.229	17	0.693	-0.619	-0.393	-0.194		
Total	-0.027	171	0.920	-0.572	-0.266	0.095		

Statistically significant effects (post-hoc test):

Standard of living: Medium vs. high (*p* < 0.05; Cohen’s d effect size = 0.0094)

We did not find any statistically significant difference in cortisol concentration between children with BMI in norm and excessive BMI in neither of the sexes (Table 4).

**Table 4:** Differences in the cortisol concentration between children with normal BMI and excessive BMI among boys and girls

Sex	BMI	N	Mean	SD	Q1	Median	Q3	Z	P	Cohen’s d
Girls	0	75	-0.086	0.869	-0.575	-0.236	0.027	0.297	0.767	0.0552
	1	18	-0.132	0.795	-0.518	-0.311	0.028			
Boys	0	58	0.102	0.940	-0.488	-0.246	0.308	1.505	0.132	0.1812
	1	20	-0.089	1.157	-0.629	-0.452	-0.180			
Total		171	-0.027	0.920	-0.572	-0.266	0.095	1.287	0.198	

BMI: Excessive (overweight or obesity) − 1; Normal − 0

When the cortisol concentration was divided into three categories: low, medium, and high and compared to the investigated components and proportions of the body, only muscle mass was statistically significant among the categories (F = 3.208; *p* = 0.043). The post hoc Tukey’s HSD test showed that the children with a medium concentration of cortisol had higher muscle mass (mean=-0.060) than those with a high concentration of cortisol (mean=-0.191) (*p* < 0.05) (Table 5). The rest of the tested body composition and proportion components did not show any statistically significantly different results (Supplementary Table 1).

**Table 5:** Comparison of the body components/proportions and cortisol concentration in Polish children

Cortisol concentration	Mean	N	SD	Q1	Median	Q3	F/H*	p
	MM (%) z-score							
Low	-0.134	44	1.058	-0.870	-0.191	0.434	3.208	0.043
Medium	-0.070	84	0.919	-0.759	-0.060	0.667		
High	0.325	43	0.866	-0.184	0.375	0.906		
Total	0.012	171	0.956	-0.707	-0.040	0.727		

Cortisol concentration: Low: < Q1; Medium: Q1-Q3; High: >Q3

Statistically significant effects (post-hoc Tukey HSD test):

MM z-score:

Low vs. Medium (*p* < 0.05; Cohen’s d effect size = 0.0646)

The cortisol concentration was negatively correlated with FM (%) and WHR z-score (R=-0.169, *p* = 0.027; R=-0.230, *p* = 0.002, respectively) and positively correlated with MM (%) (R = 0.224 *p* = 0.003).

### Stepwise forward multiple regression models

Multiple regression models were used to assess the correlation between (1) FM (%), BCM (%), MM (%), TBW (%), BMI, and WHR and (2) breastfeeding, maternal trauma during pregnancy, the standard of living, and parental education to evaluate their association with dependent variables and calculate residues for each of dependent variable.

Table 6 shows the statistically significant results of the analyses of the correlations between (1) the composition and proportion of the body and (2) environmental factors among Polish children. The first model explained the correlation between FM (%) and a high standard of living (vs. low) (Beta = 0.709; *p* = 0.033) with the control of standard of living medium (vs. low), higher paternal education (vs. basic), and breastfeeding (vs. no) explaining 3.95 % of the variability of FM (%). MM (%) was positively correlated with higher paternal education (vs. basic) (Beta = 0.618; *p* = 0.025) with the control of secondary paternal education (vs. basic), secondary maternal education (vs. basic), and breastfeeding (vs. no) explaining 6.07 % of the variability. No statistically significant correlation was found between BCM (%) and the investigated parameters. TBW (%) was negatively correlated with a high standard of living (vs. low) (Beta=-0.792; *p* = 0.016) with the control of medium standard of living (vs. low), higher and secondary paternal education (vs. basic), secondary maternal education (vs. basic), and maternal trauma during pregnancy explaining 7.02 % of the variability. BMI was positively correlated with a high standard of living (vs. low) (Beta = 0.925; *p* = 0.005) and medium standard of living (vs. low) (Beta = 0.675; *p* = 0.041) with the control of higher and secondary maternal education (vs. basic) explaining 5.95 % of the variability. The model for WHR was not statistically significantly associated with any parameter. Supplementary Table 2 shows the statistically non-significant results of this analysis.

**Table 6:** A forward stepwise multiple regression model including independent variables explaining body composition: FM (%), MM (%), TBW (%), and BMI (standardised for calendar age and sex) among Polish children

Dependent variables	Independent variables	Beta	SE	t	*p* value	Adjusted R^2^	F	*p* value	Cohen’s f^2^
FM (%) z-score	Breastfeeding - yes (vs. no)	-0.117	0.077	-0.407	0.129	0.0395	2.76	0.030	0.0411
	Paternal education higher (vs. basic)	-0.133	0.079	-0.254	0.094				
	Standard of living higher (vs. low)	0.709	0.330	2.150	0.033				
	Standard of living medium (vs. low)	0.613	0.331	1.852	0.066				
MM (%) z-score	Breastfeeding - yes (vs. no)	0.359	0.269	1.334	0.184	0.0607	2.70	< 0.033	0.0646
	Paternal education higher (vs. basic)	0.618	0.273	2.265	0.025				
	Paternal education secondary (vs. basic)	0.464	0.281	1.654	0.100				
	Maternal education secondary (vs. basic)	-0.239	0.192	-1.248	0.214				
TBW (%) z-score	Breastfeeding - yes (vs. no)	0.479	0.264	1.814	0.071	0.0702	2.85	0.008	0.0755
	Paternal education higher (vs. basic)	0.537	0.273	1.966	0.051				
	Paternal education secondary (vs. basic)	0.298	0.277	1.075	0.284				
	Maternal education secondary (vs. basic)	0.172	0.070	2.46	0.0141				
	Standard of living higher (vs. low)	-0.792	0.326	-2.433	0.016				
	Standard of living medium (vs. low)	-0.592	0.329	-1.799	0.074				
	Maternal trauma during pregnancy - yes (vs. no)	-0.314	0.237	-1.324	0.187				
BMI z-score	Breastfeeding - yes (vs. no)	-0.326	0.261	-1.252	0.212	0.0595	3.16	0.009	0.0632
	Maternal education higher (vs. basic)	-0.715	0.372	-1.924	0.056				
	Maternal education secondary (vs. basic)	-0.488	0.385	-1.265	0.208				
	Standard of living higher (vs. low)	0.925	0.323	2.864	0.005				
	Standard of living medium (vs. low)	0.675	0.327	2.063	0.041				

### Linear regression models using residues

The obtained residues for each dependent variable in the linear regression models were used to evaluate the correlations between body components and BMI and between WHR and cortisol concentration (Supplementary Table 3). The cortisol concentration was negatively correlated with FM (%) (Beta=-0.171; *p* = 0.026), explaining 2.32 % of the fat mass variability, and positively correlated with MM (%) (Beta = 0.192; *p* = 0.012), explaining 3.09 % of the muscle mass variability (Table 7). However, in both cases, the effect sizes were very low (0.0238 and 0.0389, respectively).

**Table 7:** Linear regression models explaining the variability of FM (%) and MM (%) (residues) depending on the cortisol concentration

Dependent variables	Independent variables	Beta	SE	t	*p* value	Adjusted R^2^	F	*p* value	Cohen’s f^2^
FM (%) z-score (residuals)	Cortisol concentration stand.	-0.171	0.076	-2.246	0.026	0.0232	5.04	0.026	0.0238
MM (%) z-score (residuals)	Cortisol concentration stand.	0.192	0.076	2.535	0.012	0.0309	6.42	0.012	0.0389

## Discussion

The obesity of children is a global problem [23]. Our results showed that 16.37 % of the investigated children were overweight (including 9.94 % of girls and 6.43 % of boys), and 5.85 % were obese (0.58 % of girls and 5.26 % of boys). The association between cortisol concentration and overweight is unclear. Chu et al. (2017) [24] found increased salivary cortisol concentrations among aged 4–5 years with overweight, and Veldhorst et al. (2014) [25] observed increased hair cortisol concentrations among children aged 8–12 years. Genitsaridi et al. (2018) [6] did not find increased hair cortisol concentration in obese children and adolescents aged 4–18 years. Similar results were obtained by investigations of saliva [26] and plasma among children [27]. Hill et al. (2010) [28] did not observe any association between the cortisol level and fatness among children aged 1–2 years. Our studies showed that children with decreased cortisol concentration had increased fat tissue and decreased muscle tissue. However, the cortisol concentration explained only 2.32 % of the fat mass variability and 3.09 % of the muscle mass variability, and the effect size was low in each case. Due to the diurnal variation in the cortisol concentration, it is difficult to find the exact value that affects the body composition and proportion. It is still unclear if the elevated cortisol concentration is an effect or cause of obesity. Increased levels of cortisol among obese children may be a consequence of systemic inflammation [29]. In contrary, we did not find any statistically significant difference in the cortisol concentrations between children with normal and excessive BMI. Our previous study of children aged 7–11 years did not show any association between the body components and cortisol level [15]. On the other hand, Yu et al. [7] suggested that the daily amplitude of the cortisol concentration may be more important than the cortisol concentration itself.

We found that the cortisol concentration was increased among children with a high standard of living. These findings might indicate that children from well-off families are under higher pressure, possibly due to increased expectations and increased sports activities [30]. Thus, the elevated cortisol concentrations may be caused by psychological stress and/or increased oxidative stress due to increased physical activity, which may lead to increased muscle mass among children with elevated cortisol concentration.

The parental education level was associated with body composition. Higher and secondary education among fathers was associated with increased muscle mass among the children. Similar results were previously obtained for mothers [31]. Post et al. (2018) [32] found an association between good education of the parents and increased specialization in sports disciplines of the children. Highly educated fathers may pay more attention to the sustainable development of their children by taking care of their physical activity, which may lead to increase of their muscle mass.

## Limitations

The study has some limitations. Firstly, the sample collection was only conducted once, and it is indicated by some authors that the level of the cortisol collected at the different time points may be different and preferable is the mean value of the a few daily measurements. Additionally, the body composition measurements using the BIA method may give inconsistent results [33, 34]. Thus, validation using DEXA is preferable. Moreover, the traumatic events during pregnancy were measured using a binary scale (yes or no). This method is not precise and may be subjective.

## Conclusions

We conclude that cortisol concentration is positively associated with muscle mass and negatively associated with fat mass, even when the cortisol concentration was adjusted according to environmental factors. We did not find any statistically significant differences in the cortisol concentration between children with normal BMI and excessive BMI in none of the sexes. The cortisol concentration was increased among children from well-of families.

## Supplementary information

**Additional file 1**

## Data Availability

The datasets generated during the current study are not publicly available due to law regulation but are available from the corresponding author on reasonable request.

## Abbreviations

FM (%): fat mass
MM (%): muscle mass
BCM (%): body cellular mass
TBW (%): total body water
BMI: body mass index
