# Supplementary material for: Cortisol concentration affects fat and muscle mass among Polish children aged 6–13 years
Source: BMC Pediatr. 2021 Aug 27;21:365. doi: 10.1186/s12887-021-02837-3 (PMC8394106; doi:10.1186/s12887-021-02837-3)
Supplement: Supplementary file 1 — Additional file 1 [file 12887_2021_2837_MOESM1_ESM.docx]

Supplementary data

Supplementary Table 1. Comparison of the body components/proportions and cortisol concentration standardised on sex in Polish children.

| Cortisol concentration | Mean | N | SD | Q1 | Median | Q3 | F/H* | p |
| --- | --- | --- | --- | --- | --- | --- | --- | --- |
|  | FM (%) z-score |  |  |  |  |  |  |  |
| Low | 0.119 | 44 | 0.976 | -0.563 | 0.056 | 0.804 | 1.697 | 0.186 |
| Medium | 0.053 | 84 | 0.972 | -0.602 | -0.028 | 0.698 |  |  |
| High | -0.228 | 43 | 0.889 | -0.913 | -0.210 | 0.119 |  |  |
| Total | -0.001 | 171 | 0.957 | -0.675 | -0.044 | 0.664 |  |  |
|  | BCM (%) z-score |  |  |  |  |  |  |  |
| Low | -0.075 | 44 | 1.013 | -0.613 | 0.093 | 0.489 | 0.453 | 0.637 |
| Medium | -0.008 | 84 | 0.927 | -0.627 | 0.048 | 0.585 |  |  |
| High | 0.115 | 43 | 0.933 | -0.507 | 0.382 | 0.719 |  |  |
| Total | 0.006 | 171 | 0.948 | -0.609 | 0.085 | 0.648 |  |  |
|  | MM (%) z-score |  |  |  |  |  |  |  |
| Low | -0.134 | 44 | 1.058 | -0.870 | -0.191 | 0.434 | 3.208 | 0.043 |
| Medium | -0.070 | 84 | 0.919 | -0.759 | -0.060 | 0.667 |  |  |
| High | 0.325 | 43 | 0.866 | -0.184 | 0.375 | 0.906 |  |  |
| Total | 0.012 | 171 | 0.956 | -0.707 | -0.040 | 0.727 |  |  |
|  | TBW (%) z-score |  |  |  |  |  |  |  |
| Low | -0.068 | 44 | 0.943 | -0.569 | -0.270 | 0.620 | 0.664 | 0.516 |
| Medium | -0.042 | 84 | 1.002 | -0.714 | 0.084 | 0.612 |  |  |
| High | 0.143 | 43 | 0.891 | -0.380 | 0.150 | 0.693 |  |  |
| Total | -0.002 | 171 | 0.959 | -0.591 | 0.019 | 0.637 |  |  |
|  | BMI z-score |  |  |  |  |  |  |  |
| Low | -0.044 | 44 | 0.842 | -0.583 | -0.058 | 0.148 | 4.60* | 0.100 |
| Medium | 0.161 | 84 | 1.022 | -0.707 | -0.086 | 0.714 |  |  |
| High | -0.259 | 43 | 0.856 | -0.840 | -0.346 | 0.230 |  |  |
| Total | 0.003 | 171 | 0.949 | -0.758 | -0.108 | 0.394 |  |  |
|  | WHR z-score |  |  |  |  |  |  |  |
| Low | 0.163 | 42 | 0.975 | -0.367 | 0.084 | 0.814 | 2.99 | 0.053 |
| Medium | 0.094 | 89 | 0.945 | -0.515 | 0.190 | 0.707 |  |  |
| High | -0.282 | 40 | 0.891 | -0.886 | -0.355 | 0.297 |  |  |
| Total | 0.017 | 171 | 0.950 | -0.628 | 0.031 | 0.707 |  |  |

Cortisol concentration: Low: < Q1; Medium: Q1-Q3; High: >Q3.

Statistically significant effects (post-hoc Tukey HSD test):

MM z-score:

Low vs Medium (p<0.05)

Supplementary Table 2. A forward stepwise multiple regression model including independent variables explaining the body composition: FM (%), MM (%), BCM (%), TBW (%), and body proportion (BMI and WHR) standardised for calendar age and sex among Polish children.

| Dependent variables | Independent variables | Beta | SE | t | p value | Adjusted R^2^ | F | p value | Cohen’s f^2^ |
| --- | --- | --- | --- | --- | --- | --- | --- | --- | --- |
| FM (%) z-score | Breastfeeding - yes ( vs. no) | -0.117 | 0.077 | -0.407 | 0.129 | 0.0395 | 2.76 | 0.030 | 0.0411 |
|  | Paternal education higher (vs. basic) | -0.133 | 0.079 | -0.254 | 0.094 |  |  |  |  |
|  | Paternal education secondary (vs. basic) | - | - | - | - |  |  |  |  |
|  | Maternal education higher (vs. basic) | - | - | - | - |  |  |  |  |
|  | Maternal education secondary (vs. basic) | - | - | - | - |  |  |  |  |
|  | Standard of living higher (vs. low) | 0.709 | 0.330 | 2.150 | 0.033 |  |  |  |  |
|  | Standard of living medium (vs. low) | 0.613 | 0.331 | 1.852 | 0.066 |  |  |  |  |
|  | Maternal trauma during pregnancy - yes (vs. no) | - | - | - | - |  |  |  |  |
| BCM (%) z-score | Breastfeeding - yes ( vs. no) | - | - | - | - | 0.0181 | 1.56 | 0.213 | 0.0181 |
|  | Paternal education higher (vs. basic) | - | - | - | - |  |  |  |  |
|  | Paternal education secondary (vs. basic) | - | - | - | - |  |  |  |  |
|  | Maternal education higher (vs. basic) | - | - | - | - |  |  |  |  |
|  | Maternal education secondary (vs. basic) | - | - | - | - |  |  |  |  |
|  | Standard of living higher (vs. low) | 0.521 | 0.336 | 1.551 | 0.123 |  |  |  |  |
|  | Standard of living medium (vs. low) | 0.346 | 0.336 | 1.030 | 0.304 |  |  |  |  |
|  | Maternal trauma during pregnancy - yes (vs. no) | - | - | - | - |  |  |  |  |
| MM (%) z-score | Breastfeeding - yes ( vs. no) | 0.359 | 0.269 | 1.334 | 0.184 | 0.0607 | 2.70 | <0.033 | 0.0646 |
|  | Paternal education higher (vs. basic) | 0.618 | 0.273 | 2.265 | 0.025 |  |  |  |  |
|  | Paternal education secondary (vs. basic) | 0.464 | 0.281 | 1.654 | 0.100 |  |  |  |  |
|  | Maternal education higher (vs. basic) | - | - | - | - |  |  |  |  |
|  | Maternal education secondary (vs. basic) | -0.239 | 0.192 | -1.248 | 0.214 |  |  |  |  |
|  | Standard of living higher (vs. low) | - | - | - | - |  |  |  |  |
|  | Standard of living medium (vs. low) | - | - | - | - |  |  |  |  |
|  | Maternal trauma during pregnancy - yes (vs. no) | - | - | - | - |  |  |  |  |
| TBW (%) z-score | Breastfeeding - yes ( vs. no) | 0.479 | 0.264 | 1.814 | 0.071 | 0.0702 | 2.85 | 0.008 | 0.0755 |
|  | Paternal education higher (vs. basic) | 0.537 | 0.273 | 1.966 | 0.051 |  |  |  |  |
|  | Paternal education secondary (vs. basic) | 0.298 | 0.277 | 1.075 | 0.284 |  |  |  |  |
|  | Maternal education higher (vs. basic) | - | - | - | - |  |  |  |  |
|  | Maternal education secondary (vs. basic) | 0.172 | 0.070 | 2.46 | 0.0141 |  |  |  |  |
|  | Standard of living higher (vs. low) | -0.792 | 0.326 | -2.433 | 0.016 |  |  |  |  |
|  | Standard of living medium (vs. low) | -0.592 | 0.329 | -1.799 | 0.074 |  |  |  |  |
|  | Maternal trauma during pregnancy - yes (vs. no) | -0.314 | 0.237 | -1.324 | 0.187 |  |  |  |  |
| BMI z-score | Breastfeeding - yes ( vs. no) | -0.326 | 0.261 | -1.252 | 0.212 | 0.0595 | 3.16 | 0.009 | 0.0632 |
|  | Paternal education higher (vs. basic) | - | - | - | - |  |  |  |  |
|  | Paternal education secondary (vs. basic) | - | - | - | - |  |  |  |  |
|  | Maternal education higher (vs. basic) | -0.715 | 0.372 | -1.924 | 0.056 |  |  |  |  |
|  | Maternal education secondary (vs. basic) | -0.488 | 0.385 | -1.265 | 0.208 |  |  |  |  |
|  | Standard of living higher (vs. low) | 0.925 | 0.323 | 2.864 | 0.005 |  |  |  |  |
|  | Standard of living medium (vs. low) | 0.675 | 0.327 | 2.063 | 0.041 |  |  |  |  |
|  | Maternal trauma during pregnancy - yes (vs. no) | - | - | - | - |  |  |  |  |
| WHR z-score | Breastfeeding - yes ( vs. no) | -0.320 | 0.269 | -1.193 | 0.234 | 0.048 | 5.59 | 0.108 | 0.0504 |
|  | Paternal education higher (vs. basic) | - | - | - | - |  |  |  |  |
|  | Paternal education secondary (vs. basic) | 0.224 | 0.151 | 1.480 | 0.141 |  |  |  |  |
|  | Maternal education higher (vs. basic) | - | - | - | - |  |  |  |  |
|  | Maternal education secondary (vs. basic) | - | - | - | - |  |  |  |  |
|  | Standard of living higher (vs. low) | - | - | - | - |  |  |  |  |
|  | Standard of living medium (vs. low) | - | - | - | - |  |  |  |  |
|  | Maternal trauma during pregnancy - yes (vs. no) | - | - | - | - |  |  |  |  |

Supplementary Table 3. Linear regression models explaining the variability of BCM (%), TBW (%), BMI, and WHR (residues) depending on the cortisol concentration (standardised on collection hour).

| Dependent variables | Independent variables | Beta | SE | t | p value | Adjusted R^2^ | F | p value | Cohen’s f^2^ |
| --- | --- | --- | --- | --- | --- | --- | --- | --- | --- |
| BCM (%) z-score (residuals) | Cortisol concentration stand. | 0.050 | 0.078 | 0.634 | 0.527 | <0.001 | 0.40 | 0.527 | <0.001 |
| TBW (%) z-score (residuals) | Cortisol concentration stand. | 0.109 | 0.075 | 1.449 | 0.149 | 0.0064 | 2.10 | 0.149 | 0.0064 |
| BMI z-score (residuals) | Cortisol concentration stand. | -0.093 | 0.075 | -1.230 | 0.220 | 0.0030 | 1.51 | 0.220 | 0.0030 |
| WHR z-score (residuals) | Cortisol concentration stand. | -0.142 | 0.078 | -1.833 | 0.069 | 0.0137 | 3.36 | 0.069 | 0.0139 |
